# Editorial: Implicit social cognition: malleability and change

**DOI:** 10.3389/fpsyg.2024.1475986

**Published:** 2024-09-11

**Authors:** Maddalena Marini, Janice Sabin, Brian O'Shea, Michelangelo Vianello

**Affiliations:** ^1^Department of Psychology, University of Campania “Luigi Vanvitelli”, Caserta, Italy; ^2^Department of Biomedical Informatics and Medical Education, University of Washington, School of Medicine, Seattle, WA, United States; ^3^School of Psychology, University of Nottingham, Nottingham, United Kingdom; ^4^Department of Political Science, Centre for the Experimental-Philosophical Study of Discrimination, Aarhus University, Aarhus, Denmark; ^5^Department of Philosophy, Sociology, Education and Applied Psychology, University of Padova, Padova, Italy

**Keywords:** implicit attitudes, perspective-taking, anxiety induction, virtual reality experiences, parental influences, individuation training, linguistic cues, mindfulness practices

## Introduction

Implicit attitudes can be defined as automatic evaluations of a person, group, or concept as opposed to explicit attitudes which involve deliberate introspection and controlled evaluative judgment (Corneille and Hütter, 2020; Gawronski et al., 2020; Greenwald and Banaji, 1995). Various measures have been developed to capture implicit attitudes, with the most popular being the Implicit Association Test (IAT; Greenwald et al., 1998, 2022; Nosek et al., 2011). Research has shown that these measures can predict important behaviors beyond estimates of explicit attitudes (Axt et al., 2024; Kurdi et al., 2019) and that, contrary to early theories (Kurdi and Charlesworth, 2023), contextual factors at the macro-level (i.e., societal factors and changes; Charlesworth and Banaji, 2019; Marini et al., 2013; Ofosu et al., 2019; Rauf et al., 2022) and the micro-level (i.e., online and lab studies; Blair, 2002; Lai et al., 2014, 2016; Marini et al., 2012, 2018) can manipulate and change their expression/detection.

By integrating findings from diverse research domains, this Research Topic aims to synthesize and articulate the intricate intersections between implicit attitudes, social dynamics, and intervention strategies, illuminating pathways toward fostering greater understanding and inclusivity in our increasingly diverse societies. The eight manuscripts included in this Research Topic and described in more detail below have explored the impact of diverse factors, including (1) perspective-taking, (2) anxiety induction, (3) virtual reality experiences, (4) parental influences, (5 and 6) individuation training, (7) linguistic cues, and (8) mindfulness practices, on attitudes and behaviors. Only the linguistic cue manuscript (7) used the Evaluative Priming task (Fazio et al., 1986), while the remaining used an IAT and/or one of its variants. These studies collectively offer nuanced insights, unveiling the complex mechanisms underlying human interaction and implicit attitudes.

### Perspective-taking

Skorinko et al. ran six experiments exploring gender and racial attitudes where participants believed they would interact with a partner and alignment between attitudes was assessed. Before each interaction, some participants were primed with a perspective-taking mindset and given information about their ostensible partner's views. The authors demonstrated that perspective-taking primes consistently influenced explicit attitudes, yet failed to impact implicit attitudes. Demand characteristics might have played a more influential role on the explicit level, which implicit measures are less susceptible to. These results call for further exploration into the nuanced mechanisms underlying implicit attitudes and the role of perspective-taking in shaping them.

### Anxiety induction

Müller et al. investigated if non-political anxiety (electric shock) could affect political attitudes. Participants were randomly assigned to have their political attitudes assessed either under threat (shock) or when safe (no shock threat). Psychometric and physiological data confirmed successful state anxiety induction, but this induction did not alter implicit and explicit political attitudes. This research challenges prior assumptions about the relationship between anxiety and political ideology, highlighting the importance of contextual factors (political context of threat) in understanding the impact of threats on ideology. The findings also raise the question of whether trait anxiety, as opposed to state anxiety induced in this study, might play a more significant role in influencing implicit and explicit political attitudes, suggesting a direction for future studies to explore.

### Virtual reality experiences

Marini and Casile explored how white participants embodied in either a Black or White Virtual Reality (VR) avatar, impacted their implicit racial bias. The Black avatar condition showed a reduction in participants' implicit pro-White/anti-Black attitudes compared to their baseline score, but only when they could see their virtual body from first-person and reflected perspectives. This suggests that the ability to see one's virtual body in a mirror/multiple perspectives may be crucial for the effectiveness of VR interventions in ameliorating implicit biases.

### Parental influences

Lin et al. explored the relationship between parents' education level and the gender role characteristics of ideal mates among college-aged Chinese participants, considering the moderating role of urban-rural residence. Across two studies they showed that higher parental education levels were linked to female students preferring mates with high-femininity, low-masculinity traits. For male students, higher parental education levels were associated with explicit preferences for high-masculinity, low-femininity traits. The study concluded that parents with higher educational attainment might raise children who favor partners with non-traditional gender roles.

### Individuation training relating to multiple vs. single other-race targets

Qian et al. investigated whether differentiating among multiple (four Black instructors) or a single Black individual for 2 min is optimal for reducing implicit racial bias in 4-to-6-year-olds. Using a child-friendly IAT, they showed a reduction in implicit bias against Black people only in the differentiation condition, indicating that learning to distinguish among multiple other-race individuals is critical for reducing children's implicit racial bias.

### Individuation training relating to perceived controllability of group membership

Across six studies, Rubinstein et al., addressed the moderating impact of the perceived controllability of weight and religious social group membership on bias perception, finding no supportive evidence. However, individuating information was consistently effective at shifting implicit and explicit scores toward targets from existing social groups, but results were inconsistent for novel social groups. Overall, individuation was a robust and promising means of reducing implicit biases for existing social groups.

### Linguistic cues

Hauser and Schwarz explored how neutral concepts, when frequently paired with valenced words, may foster implicit biases. They discovered that, unlike unpaired neutral primes, neutral primes paired with valenced words influenced participants' evaluations similarly to strongly valenced primes. This suggests that the implicit associations participants formed were driven not just by the direct meaning of words but by the consistent contexts in which these words appear.

Two studies found support for the causal embedding hypothesis, indicating that the language we encounter in daily life—specifically, the collocation patterns of words—can embed societal biases into our cognition. This means that linguistic biases may not only reflect societal biases but also play an active role in perpetuating them. They suggest that “anyone learning a culture's language may unwittingly learn that culture's implicit biases”.

### Mindfulness practices

Williams and Polito conducted two experiments to test if brief mindfulness meditation could reduce implicit age attitudes and sunk-cost decision-making (investment based on cumulative prior investment) bias, including increasing organizational citizenship behaviors (OCB; voluntary actions that benefit the organization and its members beyond formal job duties) at work. They showed that while mindfulness meditation significantly increased the intention to perform OCB, it did not reduce implicit age attitudes and sunk cost bias. Further research is needed to elucidate the effectiveness of meditation in bias reduction in professional environments.

## Perspectives and future directions

Moving forward, further exploration into the mechanisms underlying implicit biases is warranted and will greatly benefit from multifaceted and multidisciplinary approaches as evidenced by the diverse methodologies and theoretical frameworks presented in this Research Topic. Moreover, it is crucial that we replicate effects reported in this Research Topic, especially for studies with relatively low sample sizes, and ensure key findings generalize, particularly through using more diverse samples. Continued efforts are needed to elucidate the efficacy of interventions in mitigating biases in real-world settings (i.e., healthcare, education, and the workplace). These efforts are essential for making tangible progress in reducing implicit biases and can provide valuable insights into their feasibility and impact, ultimately contributing to more equitable and inclusive practices across society.
